# Who makes health governance decisions at the local level? A cross sectional study from primary health facilities in Tanzania

**DOI:** 10.1002/hsr2.1691

**Published:** 2023-11-05

**Authors:** Anosisye M. Kesale, Ally Kinyaga, Eliza Mwakasangula, Henry Mollel, Seif S. Rashid

**Affiliations:** ^1^ Department of Local Government Management, School of Public Administration and Management Mzumbe University Morogoro Tanzania; ^2^ Center for Reforms Innovation, Health Policies and Implementation Research (CeRIHI) Dodoma Tanzania; ^3^ Department of Public Service and Human Resource Management, School of Public Administration and Management Mzumbe University Morogoro Tanzania; ^4^ Department of Health System Mzumbe University Mbeya Tanzania; ^5^ PATH, Data Use Partnership Center of Digital and Data Excellence Dar Es Salaam Tanzania

**Keywords:** decision making, direct health facility financing, fiscal decentralization, governance, universal health coverage

## Abstract

**Background and Aims:**

Lower‐ and middle‐income countries have decentralized decision‐making at the community level, as well as community governance structures, to encourage community participation in governance processes, particularly in primary healthcare (PHC). In Tanzania, decentralization resulted in the establishment of Health Facility Governing Committees (HFGCs) to encourage community participation in the governance of primary health facilities to improve the quality and responsiveness of health service delivery. Nonetheless, despite the presence of HFGCs, PHC delivery remains ineffective and of poor quality. It is unclear who makes governance decisions at PHC facilities to ensure that services delivered are of expected quality and respond the community's needs, tastes, and preferences. This paper aims to assess the perspectives of members of the HFGC on who make governance decision in the context of fiscal decentralization.

**Design and Methods:**

A cross‐section design was used to collect both quantitative and qualitative data. A four‐multistage sampling technique was adopted to selects regions, council, health facilities, and HFGC members. Respondents who participated in structured questionnaire responses were chosen using proportional sampling, whereas those who participated in in‐depth interviews and Focus Group Discussions were chosen using purposive selection. The data was analyzed descriptively and thematically.

**Results:**

The study revealed that HFGCs members perceive that governance decisions in primary health facilities are primarily made by the health facility management, and later are presented in HFGCs. As such, HFGCs are used a passively used to justify participation in decision that was already made by the management, which contradict with the principal of decentralization that emphasizes community participation on fiscal decisions.

**Conclusion:**

Decentralization of PHC facilities does not guarantee the participation of community members in fiscal decision of their respective primary health facilities through HFGCs. HFGC is passively used governance structure to substitute community participation in primary health facilities' fiscal decisions. Enforcement mechanisms are required to facilitate effective community participation.

## 1 INTRODUCTION

Low and Middle‐Income Countries (LMICs) are experiencing extraordinary increases in healthcare funding from both development aid and government spending.^1^ Despite the fact that budget levels have a significant impact on healthcare system performance, governance is recognized for playing a vital role in improving healthcare system performance and achieving Universal Health Coverage (UHC).^2^, ^3^, ^4^ Governance is crucial in primary healthcare (PHC) for facilitating operations, accountability, and the engagement of multiple stakeholders in service provision. In this work, we define governance as the process by which communities or institutions make decisions to improve the health of the population.^5^, ^6^, ^7^ The health systems reforms guided by principal of decentralization by devolution involved the transfer of administrative, decision‐making, fiscal powers and responsibilities from the central level governance structures or higher ties to the local level governance structures. The underlying assumptions was that local‐level structures are well‐positioned to encourage community participation in the primary healthcare.^8^, ^9^ Inspired by the Alma Ata Declaration, community members' engagement is supposed to improve service quality, equity in service distribution, and nurture the relationship between facility staff and community, as well as boost service responsiveness to users or communities.^10^, ^11^ In the context of decentralization, different names have been assigned to governance structures. For instance, in Tanzania as the governance structure at primary facility levels is known as Health Facility Governing Committees (HFGCs).^9^, ^12^, ^13^

In several LMICs, community governance structures are granted decision‐making powers in the governance of primary healthcare facilities. The HFGCs, which are made up of community representatives who are typically chosen by their communities, have been given responsibility for making key governance decisions. Their duties focus on controlling operations and management, articulating community interests, ensuring that community decisions are carried out, mobilizing facility resources, employing and dismissing facility health staff, allocating facility funds, planning and budgeting for the facility, and raising facility funds.^14^, ^15^, ^16^, ^17^, ^18^ By letting local users shape the services, HFGCs are projected to increase allocative efficiency, technical efficiency, cost‐consciousness at the local level, creativity in service delivery, service quality, and equity.^8^, ^19^ HFGCs are, therefore, expected to supervise service delivery, articulate community interest, capture community health challenges, and bring them to facilities to find solutions, plan health interventions that would suit community interest, and allocate funds to interventions that matter to the community.^20^, ^21^

Although some countries have made positive comments about community involvement, many LMIC countries still have primary healthcare facilities that are characterized by poor governance and poor health outcomes despite having community governance structures in place devolved with decision‐making powers and responsibilities.^4^, ^22^, ^23^, ^24^, ^25^ Evidence suggests that community involvement has been extremely low and passive, as well as members of HFGCs have not been showing up to committee meetings, and when they do, very few have been voicing up.^4^, ^23^, ^24^ Limited fiscal powers and awareness on their roles are cited as major factors obstructing their meaningfully participation.^23^, ^25^, ^26^ In some other countries community participations have been concluded to be symbolic.^14^, ^27^

HFGCs in Tanzania were established in 1999 and assigned specific powers and responsibilities to perform. The functionality of HFGCs in Tanzania has been demonstrated empirically to be extremely low, and service delivery has been characterized by subpar health results.^12^, ^20^, ^28^, ^29^ The government chose to pursue fiscal decentralization through Direct Health Facility Financing (DHFF) to enhance PHC health service delivery and strengthen community ownership and autonomy.^30^, ^31^ The DHFF procedure involves the direct deposit of facility funds into facility accounts from several sources. Before DHFF, funds for primary healthcare facilities were deposited into district or council accounts from various sources, and all financial management of the funds was handled by the Council Health Management Team (CHMT) and the District Medical Officer.^12^, ^30^ Decisions made by the HFGC were heavily influenced by the council's agenda, which eliminated the HFGCs desire to engage in decision‐making and led to its eventual passivity. This is because decentralization in Tanzania began by transferring powers and responsibilities from the national to the district or council level. At PHC facility, only political and administrative duties were decentralized, preventing many decisions from being made and implemented relevant facility actors.^25^ According to the literature, fiscal decentralization significantly aids other aspects of decentralization, such as administrative and decision‐making, in functioning well or having significance. Fiscal decentralization entails transfer of the expenditure and revenue responsibilities to the subnational levels.^32^, ^33^ The choice to adopt the DHFF strategy was made to overcome significant obstacles like funding delays and misappropriation, prolonged procurement processes, limited decision‐making space, and a lack of ownership in the execution of various programs and interventions at lower‐level health facilities. The Governing Committee (HFGC) is given tasks for overseeing the execution of the facility operations under fiscal decentralization through DHFF.^30^

However, literature caution that the decentralization results have been mixed as granting decision space to communities does not guarantee them to use that space. The design and implementation of the decentralization has always been a case on determining the gains.^8^ In Tanzania, there scant of information on whether HFGCs members participate in decision‐making under fiscal decentralization. This study was determined to assess who make governance decision at the PHC under the DHFF context in selected primary health facilities implementing DHFF.

## 2 STUDY SETTING

Healthcare system in Tanzania is shaped like a pyramid. A community is at the bottom of the health system, followed by dispensaries, health centers, district council hospitals, regional referral hospitals, zonal hospitals, and finally the national hospital is at the top. The development and administration of the nation's health sector policies are under the control of two ministries. Health resource mobilization and policy formulation are the responsibilities of the Ministry of Health, whose name is subject to change over time. Additionally, this ministry is in charge of overseeing the delivery of healthcare services at the national hospital, specialized hospitals, zonal hospitals, and regional referral hospitals. The interpretation of policies and coordination of the implementation of health policies are under the President's Office—Regional Administration and Local Government (PO‐RALG), which is also in charge of managing service delivery at the community level (through mobile and outreach services, dispensaries, health centers, and district hospitals). The Regional Health Management Team, a specific decentralized structure, oversee the delivery of health services at the regional level. The CHMT, a structure for managing council health, is in charge of managing health services at the district council level. All district‐level health program implementation falls under the purview of this team. The CHMT is in charge of ensuring that DHFF is implemented in accordance with its design and is in charge of offering technical support to primary healthcare facilities regarding DHFF implementation. A primary healthcare facility called a health center is located at the ward level. The Health Facility Management Team and the HFGC, who oversee the implementation of the facility's health services, are in charge of managing health services at this level. All transactions at healthcare facilities are approved by the HFGCs, and they are essential to the inspection of incoming medical supplies before they are distributed. They participate in the planning and budgeting process for the healthcare facility and oversee the construction or minor renovation of a few structures. The HFGCs have also recently been placed at the center of health reform by proponents of performance‐based financing, who anticipate that it will hold healthcare providers accountable for the performance of their medical facilities.

Indeed, Healthcare financing in Tanzania is mainly funded by the government, through Ministry of Finance. Other fundings come from the development partners NGOs, prepayment scheme (Community health fund, private insurance scheme) and out‐of‐pocket contribution or money paid directly to health providers by client after accessing health services.^12^, ^34^ The health sector is among of the largest sector in Tanzania, for instance, in the fiscal year 2019/20, the sector was allocated a total Tanzanian shilling 2.21 trillion which is 6.7% of the total nation budget and 1.5% of the Gross Domestic Product.^29^

From 2015, the government of Tanzania started to conduct Star Rating Assessment to all primary healthcare facilities in the country. The primary goals of the star rating assessment were to analyze the effectiveness of medical facilities and offer suggestions for enhancement. In the star rating assessment, first, scoring was carried out in a way that the rating was based on the score of the minimum scoring domain rather than the total or average marks (0%–19% no stars, 20%–39% one star, 40%–59% two stars, 60%–79% three stars, 80%–89% four stars, and 90%–100% five stars). The improvement initiative's second goal was to have 80% of primary healthcare facilities receive a three‐star rating by 2017–2018, establishing a minimum requirement for healthcare facilities that is comparable to a three‐star facility. At the time, the belief was that all facilities with zero stars should be shut down because they would be dangerous and unfit for providing services.^13^ One of the areas which was assessed and rated during assessment was social accountability in which the performance of HFGCs in each primary health facility was assessed and scores were provided for each facility. These scores were the base of selecting research sites for this study. The selection of the health facilities based on their performance is supported by the conclusion and argument made by McCoy et al.^35^ that when the performance of the health facility is high tend to reflect the performance of the HFGCs or mostly the performance of facility governance committees such as HFGCs tend to be high too. The essence of selecting the regions, districts, facilities that had high and low performance was to ascertain if facility performance corresponds with the performance of HFGCs participation in making decisions regarding facility operations. Therefore, in this study, all facilities which scored rating from three stars and above are regarded as high‐performing health facilities while those which scored below three stars were categorized as low‐performing facilities. Based on the argument made by McCoy, this study assumes that the facility under the high‐performance category also have a likelihood of having high HFGCs participation in decision‐making while the facility under the low‐performance category are likely to have low participation of HFGCs in decision‐making.

## 3 MATERIALS AND METHODS

### 3.1 Study design

A cross‐sectional design was employed to collect both qualitative and quantitative data at the same time between February and April 2021 to ascertain who participate in making governance decisions in a particular health facility under DHFF context.

### 3.2 Sample size and sampling techniques

In this study, both probability and nonprobability were used sampling techniques to choose study subjects. Four four‐stage multiple sampling procedure was employed to select research sites based on the levels and organization of health system in Tanzania. Star rating assessment report findings of 2018, the year when DHFF implementation started was used to select facilities from both high and low‐performing regions and districts. Therefore, four‐stage multistage sampling technique was applied in selecting four regions. At stage one two regions Kilimanjaro and Mbeya were selected because had facilities with high performance during the star rating assessment and the other two regions Ruvuma and Songwe had majority of health facilities with low performance. In stage two, two councils were selected from each selected region in stage one, in which the inclusion criteria was a council which had majority of health facilities with high performance and a council which had majority of health facility with low performance in the region. In stage three, four health facilities were selected from each selected council in stage two, the health facilities were selected based on performance of the given health facility in the council as per star rating assessment conducted in 2018 and the location of the health facility in the council. At this stage, a dispensary and health center which had high performance in the council and those which had low performance were purposively selected. We also considered whether the facility is from urban or rural areas within the council. Table 1 shows the sampling process and techniques used.

**Table 1:** Sampling process and sampling techniques.

Stage	Respondent	Sampling procedure	Inclusion criteria
First stage	Four (4) regions selected. Kilimanjaro, Mbeya, Ruvuma, and Songwe	Purposive	Performance of the region, Zonal representation—regions with high and low‐performing facilities
Second stage	Eight LGAs selected; two LGAs from each of the above‐selected regions	Purposive	Performance of the LGA in star rating assessment, nature of the LGA (Urban and Rural)
Stage three	32 health facilities were selected from all (eight) councils. Two health centers and two dispensaries from each LGA because they all implement DHFF	Stratification of health facilities into Health centers and Dispensaries Purposive selection of health centers and dispensaries	Performance of health facility (high and low‐performing health center and dispensary), Location of the facility within the LGA (Diversity)
Stage four	288 HFGC members; nine members from each selected health facility	Proportional sampling Purposive for interview and FGDs	Members of the HFGC

Abbreviations: DHFF, Direct Health Facility Financing; FGD, Focus Group Discussion; HFGC, Health Facility Governing Committee; LGA, Local Government Authority.

At stage four, the representatives from HFGCs were obtained by applying the proportion sampling technique, the formula assumed 95% confidence of level and *p* at 0.5. Therefore, according to the techniques, the size of HFGCs members required was 288, however, the response was 280 respondents.

### 3.3 Recruitment of participants for interviews and focus group discussion (FGD)

Chairpersons of all participating HFGCs were chosen for in‐depth interviews, while the rest of the members of the HFGCs were recruited for FGDs.

### 3.4 Data collection methods and techniques

#### 3.4.1 Data collection

A systematic, closed‐ended questionnaire was developed to collect quantitative data from HFGCs members. Open Data Kit software was used to develop the database and data collection tools (ODK). To ensure speed, accuracy, and reducing cost in collecting data, Mobile Data Collection (MDC) strategy was employed in collecting both quantitative and qualitative data.^30^ Four research assistants were recruited and trained for 3 days in how to conduct the interviews and on MDC skills and methodologies. The abilities they learned were then put to the test in a few facilities outside the study region. Through the ODK portal, the researcher received the collected data. All research assistants used tablets with GPS sensors to ensure that the selected study health facilities were the ones visited. A total of 280 out of 288 HFGCs members participated with 97% response rate.

We used in‐depth interview questionnaires and FGDs guiding templates to collect qualitative data. A total of 14 in‐depth interviews with HFGC Chairpersons from selected health facilities were undertaken to learn about the experiences of HFGC members in making decisions on governance of health facility operations in each of the functions allocated to the HFGCs under the DHFF. A total of 14 FGDs involving HFGC members each with six to nine people were conducted. After saturation was attained, the number of focus groups and interviews was reduced. Interviews and FDGs were done in rooms where participants could speak freely. Both quantitative and qualitative data were used so that to deepen understanding on who make governance decisions at the local level. Furthermore, qualitative data was used to explain the quantitative results as to why and how governance decisions are made at the local level, particularly at the PHC.^36^

#### 3.4.2 Data analysis

Quantitative data were analyzed using descriptive statistics and the results are presented in form of tables, for categorical variables frequency and percent were reported and for continues variables mean and standard deviation were reported. Statistical Product and Service Solution (version 25) were used in data cleaning and Statistical Analysis System (version 9.4) were used in data analysis. Qualitative data was assessed using a five‐step analysis approach. Patterns and themes that surfaced throughout data collection were accommodated by the procedure. Since audio recordings of the acquired data were made, the analysis procedure began with the transcription of the audio into notes from in‐depth interviews and FGDs. To identify the differences and similarities among different types of primary health centers, the data were then coded based on keywords pertaining to the functionality of HFGCs. The study's goal‐related theme categories were then found to help understand the data and how they relate to one another.

### 3.5 Ethical approval and informed consent

This study was conducted in accordance with the principles of the Declaration of Helsinki. All methods were carried out in accordance with relevant guidelines and regulations. Ethical approval for the study was obtained/sought by the Institutional Review Boar (IRB) of the Sokoine University of Agriculture. The IRB with the number SUA/ADM/R. 1/8/668 was sought from the Sokoine University of Agriculture. The permit was then submitted to the PO‐RALG to be permitted to research local government authorities. PO‐RALG offered a permit with registration number AB.307/323/01 to allow the research in the selected regions. Informed consent was obtained in writing from all study participants.

## 4 RESULTS

### 4.1 Demographic characteristics of respondents

A total number of 280 HFGCs members aged between 23 and 76 with mean age of 46.53 ± 12.43 participated in this study. Region wise, 31.19% of respondents were from Kilimanjaro, 22.77% respondents from Mbeya, 21.78 from Songwe, and 24.26 from Ruvuma region. A total of 57.43% (*n*) of respondents were from dispensary, while 42. 57 from health centers. In terms of position, 12 chairpersons, 12 Secretaries of HFGCs and *n* (73%) were HFGCs members of the 14 recruited HFs participated in the study. A total of 50% of the respondents were male. Near a quarter of study participants had attained above secondary certificate, Diploma, and degrees, while just above half of them had primary school education. More detained demographic characteristics are as outlined in Table 2.

**Table 2:** Demographic characteristics of respondents.

Variable	Frequency	Percent
Region		
Kilimanjaro	93	33.21
Mbeya	69	24.64
Songwe	54	19.29
Ruvuma	64	22.86
Type of health facility		
Dispensary	161	57.50
Health center	119	42.50
Position		
Chairperson	43	15.36
Secretary or facility in charge	34	12.14
Member of the HFGC	203	72.50
Age		
23–30	32	11.43
31–45	100	35.71
46–60	107	38.21
61–76	41	14.64
Sex		
Male	139	49.64
Female	141	50.36
Education level		
Primary	150	53.57
Secondary	64	22.86
Certificate	24	8.57
Diploma	30	10.71
Advanced diploma	5	1.79
University degree	7	2.50

Abbreviation: HFGC, Health Facility Governing Committee.

### 4.2 Perception of HFGC members on who makes governance decisions in primary health facilities that had low‐performance star rating scores

The HFGC members were required to show the perception on who makes governance decision in the PHC facilities. Six options were made available to them to indicate who makes decisions at the facility level. Table 3 shows the perception of all participants from both facilities that had high and low performance in the star rating assessment on who make governance decisions in their facilities.

**Table 3:** General perception of HFGC on who make governance decision among health facilities.

Statement	Don	HFGcm	HFinch	Chpsec	inchcob	Village
Prepare facility plans according to community needs	3 (1.07%)	124 (44.29%)	97 (34.64%)	24 (8.57%)	22 (7.86%)	10 (3.57%)
Managing facility funds	4 (1.43%)	51 (18.21%)	174 (62.14%)	22 (7.86%)	15 (5.36%)	14 (5.00%)
Procuring health equipment, drugs, and services	6 (2.14%)	24 (8.57%)	177 (63.21%)	33 (11.79%)	28 (10.00%)	12 (4.29%)
Promote health workers' affairs and Control health workers' discipline	9 (3.21%)	43 (15.36%)	160 (57.14%)	22 (7.86%)	35 (12.50%)	11 (3.93%)
Management of facility resources	2 (0.71%)	60 (21.43%)	166 (59.29%)	22 (7.86%)	15 (5.36%)	15 (5.36%)
Mobilization of facility finances from different sources	1 (0.36%)	50 (17.86%)	172 (61.43%)	24 (8.57%)	14 (5.00%)	19 (6.79%)
Constructing facility infrastructures and renovating the existing	2 (0.71%)	53 (18.93%)	165 (58.93%)	24 (8.57%)	12 (4.29%)	24 (8.57%)
Discussing the challenges confronting the community	2 (0.71%)	59 (21.07%)	170 (60.71%)	13 (4.64%)	6 (2.14%)	30 (10.71%)

*Note*: Don = *I don't know*. HFGcm = *Members of HFGC jointly make a decision*. HFinch = *Health facility in‐charge together with facility management team make a decision*. Chpsec = *Chairperson and secretary of the committee make a decision*. Inchcob = *The facility in charge in collaboration with other health workers make a decision*. Village = *Village/mtaa social committee in consultation with the facility in charge make a decision*.

Abbreviation: HFGC, Health Facility Governing Committee.

Table 4 presents the participants perception on who governance make decisions in public primary health facilities that had low performance in 2018.

**Table 4:** Responsibility of making decisions among low‐performance health facilities.

Statement	Don	HFGcm	HFinch	Chpsec	inchcob	Village
Prepare facility plans according to community needs	2 (1.49%)	59 (44.03%)	47 (35.07%)	13 (9.70%)	9 (6.72%)	4 (2.99%)
Managing facility funds	2 (1.49%)	20 (14.93%)	86 (64.18%)	11 (8.21%)	8 (5.97%)	7 (5.22%)
Procuring health equipment, drugs, and services	4 (2.99%)	13 (9.70%)	75 (55.97%)	26 (19.40%)	10 (7.46%)	6 (4.48%)
Promote health workers' affairs and Control health workers' discipline	7 (5.22%)	18 (13.43%)	83 (61.94%)	12 (8.96%)	8 (5.97%)	6 (4.48%)
Management of facility resources	0 (0.00%)	32 (23.88%)	80 (59.70%)	11 (8.21%)	5 (3.73%)	6 (4.48%)
Mobilization of facility finances from different sources	0 (0.00%)	21 (15.67%)	86 (64.18%)	14 (10.45%)	3 (2.24%)	10 (7.46%)
Constructing facility infrastructures and renovating the existing	1 (0.75%)	21 (15.67%)	86 (64.185)	13 (9.70%)	5 (3.73%)	8 (5.97%)
Discussing the challenges confronting the community	0 (0.00%)	23 (17.16%)	90 (67.16%)	8 (5.97%)	1 (0.75%)	12 (8.96%)

*Note*: Don = *I don't know*. HFGcm = *Members of HFGC jointly make a decision*. HFinch = *Health facility in‐charge together with facility management team make a decision*. Chpsec = *Chairperson and secretary of the committee make a decision*. Inchcob = *The facility in charge in collaboration with other health workers make a decision*. Village = *Village/mtaa social committee in consultation with the facility in charge make a decision*.

In the high‐performance facilities, the responsibility of who makes decision were assessed and results are presented in Table 5.

**Table 5:** Responsibility of making decisions among high‐performance health facilities.

Statement	Don	HFGcm	HFinch	Chpsec	inchcob	Village
Prepare facility plans according to community needs	1 (0.68%)	65 (44.52%)	50 (34.25%)	11 (7.53%)	13 (8.90%)	6 (4.11%)
Managing facility funds	2 (1.37%)	31 (21.23%)	88 (60.27%)	11 (7.53%)	7 (4.79%)	7 (4.79%)
Procuring health equipment, drugs, and services	2 (1.37%)	11 (7.53%)	102 (69.86%)	7 (4.79%)	18 (12.33%)	6 (4.11%)
Promote health workers' affairs and Control health workers' discipline	2 (1.37%)	25 (17.12%)	77 (52.74%)	10 (6.85%)	27 (18.49%)	5 (3.42%)
Management of facility resources	2 (1.37%)	28 (19.18%)	86 (58.90%)	11 (7.53%)	10 (6.85%)	9 (6.16%)
Mobilization of facility finances from different sources	1 (0.68%)	29 (19.86%)	86 (58.90%)	10 (6.85%)	11 (7.53%)	9 (6.16%)
Constructing facility infrastructures and renovating the existing	1 (0.68%)	32 (21.92%)	79 (54.11%)	11 (7.53%)	7 (4.79%)	16 (10.96%)
Discussing the challenges confronting the community	2 (1.37%)	36 (24.66%)	80 (54.79%)	5 (3.42%)	5 (3.42%)	18 (12.33%)

*Note*: Don = *I don't know*. HFGcm = *Members of HFGC jointly make a decision*. HFinch = *Health facility in‐charge together with facility management team make a decision*. Chpsec = *Chairperson and secretary of the committee make a decision*. Inchcob = *The facility in charge in collaboration with other health workers make a decision*. Village = *Village/mtaa social committee in consultation with the facility in charge make a decision*.

### 4.3 Qualitative results

#### 4.3.1 Experience of the HFGC members on the decision‐making under the DHFF context

Several themes emerged to be common to many respondents who participated in the study through in‐depth interviews and FGDs. The respondents were on the view that decision‐making at the primary healthcare facilities under DHFF context has been around financial decisions, procurement decisions, planning, and budgeting decisions. Informational reports decisions and community challenges resolutions, human resources decisions.

#### 4.3.2 Financial decisions

Respondents from all HFGCs had different opinions regarding the extent they have been participating in making decision about financial issues. Some of the members both from high and low‐performing health facilities responded that they have been making decisions concerning increasing revenue and expenditure and innovating new sources of funds to the health facility, one of the response was “Listen … here at our facility we as committee we are very strictly on deciding on how to use the collected funds to activities that improve service” (HFGC Chairperson‐high‐performing facility) Other said that they have been making decision on amount to be used in some facility operation such as construction activities. On the other hand, some respondents mostly being from lower performing health facilities said that they have been infrequently engaged in making fiscal decision as they have been getting instruction from health facility in charge on how funds need to be used basing on the national priorities, … *I think the issue of finance is under the health facility in charge, for us committee what we do is just to approve the use of the finances* (FGD response‐low‐performing facility).

Respondents were on the opinion that financial regulation have been limiting flexibility on making decisions basing on the current situation because they have limited amount or percentage to be used in some activities such as medicine. Respondents had this to say.

#### 4.3.3 Procurement decisions

Members of HFGC had a mixed reaction on the extent they have been participating in decision‐making pertaining to procurement issues. While most of members from high‐performing health facilities responded that they have been making decision regarding approving procurement plans and deciding on the medicine and medical commodities that are to be procured based on the need of the facility as revealed in some of their response … *We make sure everything that we want to purchase first is included in the procurement plan of the facility, so we really work together with the facility in charge to identify procurement needs* (FGD‐ high‐performing facility) a good number of members from low‐performing health facilities responded that they have not been engaged as the facility management team is responsible for that purchasing issue

… however, the respondents from both low and high‐performing health facilities responded that they have been inspecting the procured goods such as building materials, medicines, and medical commodities however regarding the standard and specification of the items the medical in charges have deciding. Most of the members from low‐performing health facilities were on the view that they have not been making decisions regarding procurement because the issue need certain kind of educational level for one to be in a position to make decisions statement such as … *My friend, do you think with standard seven you can inspect or participate in making decision on profession issues like procurement? To be frankly we do not make any decisions on such matter* (FGD‐low‐performing facility).

#### 4.3.4 Planning for health facility

Regarding deciding health facilities plans, respondents stated that they have been participating in planning process even not all of the decisions which they made tend to be approved by other levels such as CHMT. Interestingly some members from low‐performing facilities said that they have been receiving directives from the health facility management on what to decide regarding facility plans. Budgetary ceiling has been claimed by respondents as impediment for their suggestion to be accommodated in the health facility plans as revealed by statement such as … *Yes, we make plans on what to be implemented by the facility in a next year, the challenge is that when the plan is forwarded to the council level some of our planned interventions are canceled* (FGD‐low‐performing facility). Even though some respondents high‐performing facilities were in the view that despite that ceiling they have been working together with the health facility management to make decisions that community challenges are priorities and solved with the same limited budget … *We and health facility in charge we are one thing, we decided together on what to implement given the resource the facility manage to mobilize* (FGD‐high‐performing facility).

#### 4.3.5 Information reports presented to the HFGCs

In Tanzania, HFGCs works through four quarterly meetings in a year, in each quarterly meeting health facility management is required to present different reports regarding the progress of health facility operations such as implementation of the facility plans. When asked what kind of decision have been making over the presented reports, some respondents from both high and low‐performing facilities said that they have been deciding over what to be done by the management, sometimes requesting a special investigation to been done …“We ensure that all reports are presented to the HFGC meetings and the from each reported issue the committee decide the way forward” (FGD‐high‐performing facility). Other respondents from low performing said that the secretary of the HFGC who health facility in charge is has been presenting very few reports especially on finance but they are not given a space to contribute anything….“In our HFGC meetings we normally don't ask questions or do anything because the health facility in charge present and explain everything” (FGD‐low‐performing health facility). In line with that, some other respondents from both low and high facilities in the view that they did not know if they are required to decide anything, and they don't know which report need to be presented.

#### 4.3.6 Overseeing health workers' performance

Overseeing and managing the performance of the health facility workers is one of the core functions mandated to the HFGCs. Majority respondents were of the opinion that major decision that they have been making regarding health workers is approving the recruitment of casual workers such as cleaners and security guards …“In our facility the major decision that we have been doing regarding health workers is to approve the recruitment of security guards and cleaners who are being paid by the facility itself” (FGD‐High‐performing facilities). Also, some respondents from the high‐performing facilities responded that they have been directing health facility in charge to take some investigation and disciplinary measures to the facility health workers who claimed by the community to mistreat patients. However, majority of respondents from both high and low‐performing facilities had different opinion as they said that it is difficult of HFGC to decide anything about health workers because the guideline does not specify how they should be acting, therefore, they have not been making any decision …“We thought health facility in charge is responsible for supervising the health workers, we as committee members we do not decide anything” (HFGC Chairperson‐Low‐performing facility).

#### 4.3.7 Construction and renovation of infrastructure

As a core functions of the HFGC, respondents said that they have been making decisions about which infrastructure to be constructed or renovated in each year. Indeed, some few respondents from both low‐ and high‐performing facilities went further by saying that they have been participating in deciding even the constructor who would effectively construct or renovate infrastructure from communities which have made them use very little cost. *We been deciding about infrastructure to be developed or renovated after thorough assessment of the facility needs* (FGD‐high‐performing facility). On the other hand, majority of respondents from both high‐ and low‐performing health facilities said everything pertaining construction and renovation of the facility are done by the health facility management but the HFGCs just approve the funds for that activity … *We do not decide about construction because that is decided by the facility management and the government, we just wait the government to say what to construct* (FGD‐low‐performing facility).

## 5 DISCUSSION

The purpose of this study was to assess HFGC members' perceptions on who makes governance decisions in primary health facilities in Tanzania from both the low and high star rating performance of 2018 report. Therefore, the study intended to determine whether fiscal decentralization through DHFF has enabled HFGCs to make their mandated governance decisions. This research revealed that contrary to expectations, decisions in components assigned or mandated to HFGCs are decided mainly by the health facility in charge and facility management within the DHFF context rather than by the HFGCs. One of the goals of implementing fiscal decentralization at PHC facilities via DHFF was to increase community autonomy and ownership the health services delivery at the PHC through increase HFGCs participation in overseeing health facilities.^12^, ^37^

Specifically, the study discovered that across the eight functional areas of HFGC, HFGC members perceive a high level of participation in planning and budgeting. The study, on the other hand, suggests that members believe that the health facility in charge and facility management team makes governance decisions in the remaining governance functional areas that HFGC members were expected to perform in primary health facilities under the DHFF context. The findings of this study indicate that there is still a long way to go until Alma Ata's objectives of making community participation at the center in planning, implementing, and monitoring of PHC services are met.^38^, ^39^ This is due to the fact that participation is very low, therefore, their contribution may be quite small. Some members were of the opinion that they had been attending meetings but were scared to voice community concerns, allowing health providers to dominate decision‐making platforms that were meant for communities.

Furthermore, the study's findings suggest that the services offered at PHC facilities are largely determined by service providers (facility management), rather than by communities deciding their own health. This is contrary to the spirit of the primary health goal as well as the UHC objectives, which call for users/communities to decide the supply of services to remove financial barriers to access, improve access, and quality.^40^ Failure to participate the community or the HFGC in overseeing service provision implies that service provision in primary health facilities may not be responding to community preferences, need and tastes, and as a result, the community may be hesitant to use services produced by Tanzania's public primary health facilities.

The study also suggests that there is a slight difference in decision‐making between primary health facilities that performed poorly and those that had high performance, because both have revealed that HFGCs only make decisions in planning and budgeting, while the facility management team makes decisions in other functional governance areas of the HFGCs. This suggests that fiscal decentralization through DHFF has not been very successful in empowering communities to feel more secured in exercising their powers and authorities in governing PHC facilities. This result supports Bossert's contention that giving communities decision‐making space through decentralization does not guarantee community participation or use of the granted space.^41^ This research confirms that, despite being offered the opportunity to make governance decisions through HFGCs, members need to be capacitated to play their roles rightly. A similar incident was experienced in Burundi, where community health committees were given fiscal powers and mandates but did not utilize them.^14^

Therefore, the study is against the McCoy et al. argument that when a facility has high performance, the performance of their HFGC tends to be high^35^ because this study suggest that high or low performance of the primary health facility is not related to the performance of HFGCs. indeed, the use of star rating assessment for which indicated the performance of primary health facilities and relating with the performance of HFGCs might not be a good factor. This is because star rating had 12 components relating with service delivery at the PHC facilities in which one of them was social accountability or performance of HFGCs. Therefore, there might be many components at the facility level which do not need HFGCs influence to operate.

In general, the finding suggests how governance structures at PHC facilities remain weak in carrying out their decentralized missions. Decentralization is still relevant at the local level such as primary healthcare facilities as advocated by the fiscal federalism theory that at the local level health services provided are specific/local based in nature, therefore, can be well executed and shaped by the local people rather than being centralized. The purpose of establishing community governance structures was to ensure that communities could participate in deciding what kind of services they want to be offered to them, influence service providers' accountability, efficacy, efficiency, and responsiveness, resulting in UHC. However, this is not the case because service providers appear to be highly powerful and bear both management and governance obligations. This may reduce health systems' accountability, effectiveness, and responsiveness in providing health services.

## 6 CONCLUSION

Building robust community governance structures is a commendable step toward achieving UHC in LMICs. This is because robust governance institutions provide assurance that critical governance decisions made by these governance organs reflect the will, preferences, and needs of the people. However, the findings of this study demonstrate that, despite significant efforts by governments, the capacity of health facility governance structures to carry out their delegated functions and mandates remains low. It calls for further research on how HFGCs be empowered to resume their mandated functions that will achieve the desired impact including improvement of PHCFs quality service delivery. This necessitates a greater focus on the qualities of members of community governance institutions to assist them in carrying out their powers and mandates.

## AUTHOR CONTRIBUTIONS

**Anosisye M. Kesale**: Conceptualization; data curation; formal analysis; investigation; methodology; project administration; resources; supervision; validation; writing—original draft; writing—review and editing. **Ally Kinyaga**: Data curation; formal analysis; methodology; software; validation. **Eliza Mwakasangula**: Conceptualization; data curation; formal analysis; funding acquisition; methodology; project administration; resources; supervision; writing—original draft; writing—review and editing. **Henry Mollel**: Conceptualization; methodology; project administration; resources; supervision; writing—original draft; writing—review and editing. **Seif S. Rashid**: Conceptualization; funding acquisition; methodology; resources; supervision; writing—original draft; writing—review and editing. All authors have read and approved the final version of the manuscript and had full access to all of the data in this study and take complete responsibility for the integrity of the data and the accuracy of the data analysis.

## CONFLICT OF INTEREST STATEMENT

The authors declare no conflict of interest.

## TRANSPARENCY STATEMENT

The lead author Anosisye M. Kesale affirms that this manuscript is an honest, accurate, and transparent account of the study being reported; that no important aspects of the study have been omitted; and that any discrepancies from the study as planned (and, if relevant, registered) have been explained.

## Data Availability

All data have been included in this manuscript.
